# Case Report: Suspected *Mycobacterium abscessus*–associated polyserositis following coronary stenting, diagnosed with the aid of metagenomic next-generation sequencing

**DOI:** 10.3389/fmed.2026.1910932

**Published:** 2026-09-09

**Authors:** Yimeng Zhou, Lin Zhang, Xiaoxia Liu, Dan Wu

**Affiliations:** 1 Department of Infectious Diseases, Shengjing Hospital of China Medical University, Shenyang, China; 2 Department of Radiology, Shengjing Hospital of China Medical University, Shenyang, China

**Keywords:** case report, metagenomic next-generation sequencing, *Mycobacterium abscessus*, non-tuberculous mycobacteria, polyserositis

## Abstract

**Background:**

*Mycobacterium abscessus* (*M. abscessus*), a rapidly growing non-tuberculous mycobacterium (NTM), rarely causes extrapulmonary disease involving more than one serosal compartment.

**Case presentation:**

An 83-year-old man developed intermittent fever about 6 weeks after coronary stent implantation. Multiple antibiotics were ineffective, and he was admitted for fever of unknown origin. Echocardiography showed newly developed pericardial adhesion with echocardiographic changes suggestive of possible early constrictive physiology. These were absent on the echocardiogram performed on the day of percutaneous coronary intervention. During admission, he underwent surgery for mechanical small-bowel obstruction, in which dense omental adhesions were found. With no prior abdominal surgery, the synchronous pericardial, pleural, and peritoneal abnormalities raised suspicion of a systemic inflammatory or infectious process. A single blood-culture draw at the outside hospital grew coagulase-negative staphylococcus, but blood cultures repeated after admission and a bone-marrow culture were negative. Blood metagenomic next-generation sequencing (mNGS) detected *M. abscessus* at very low abundance (three species-level reads, below the ≥8-read threshold), reported as a suspected organism. Temperature improved during the administration of agents with recognized activity against *M. abscessus* (linezolid, tigecycline) and relapsed during the administration of β-lactams not active against this organism. Integrating the clinical course, treatment response, and consultation from a tuberculosis specialty hospital, a clinically suspected diagnosis of *M. abscessus* infection supported by mNGS was made, although microbiological confirmation was not obtained. Temperature normalized on tigecycline and the patient’s condition improved, and he was transferred for continued anti-NTM therapy. He was subsequently lost to follow-up, and therefore, the long-term outcome is unknown.

**Conclusion:**

We report a rare presentation of suspected *M. abscessus*-associated polyserositis following coronary stenting, in which mNGS provided the etiologic clue when cultures were unrevealing. NTM should be considered in older patients with unexplained fever after cardiovascular intervention. Low-abundance mNGS results, although below reporting thresholds, may be clinically meaningful when interpreted alongside the clinical course and treatment response, while remaining open to contamination and misclassification. NTM-associated myeloperoxidase-antineutrophil cytoplasmic antibody positivity is non-specific and must be distinguished from primary vasculitis.

## 1 Introduction

*Mycobacterium abscessus* (*M. abscessus*; also designated *Mycobacteroides abscessus* under a proposed genus reassignment that has been inconsistently adopted in clinical microbiology, and hereafter referred to as *M. abscessus*) is one of the most common pathogenic species among rapidly growing non-tuberculous mycobacteria (NTM) and among the most drug-resistant mycobacteria (1, 2). NTM disease has risen worldwide over the past two decades, and *M. abscessus* is the second most common NTM pulmonary pathogen. In Scotland, the incidence of *M. abscessus* infection rose from 0.4 to 0.8 per 100,000 person-years between 2011 and 2019 (3). The organism causes pulmonary, skin and soft-tissue, osteoarticular, and disseminated infections (4). Extrapulmonary infection is often associated with surgery, trauma, or immunosuppression (1, 5).

NTM infection after cardiovascular intervention is occasionally reported but is a rare occurrence. In older patients, immunosenescence and chronic kidney disease (CKD)-related immune dysfunction may create a substrate of susceptibility (6, 7). Such dysfunction is easily overlooked when daily function is preserved. A diagnosis of *M. abscessus* is challenging: conventional culture is slow and insensitive, particularly after antibiotic exposure (8). Metagenomic next-generation sequencing (mNGS) has shown advantages for NTM (9, 10). NTM infection can also induce autoantibodies such as antineutrophil cytoplasmic antibody (ANCA), risking misdiagnosis as vasculitis (11, 12).

Here, we report an older patient with clinically suspected *M. abscessus* infection following a percutaneous coronary intervention (PCI), presenting as polyserositis with synchronous pericardial, pleural, and peritoneal abnormalities. Throughout this report, polyserositis denotes synchronous abnormalities of more than one serosal compartment rather than microbiologically confirmed infection of each surface. We discuss how the diagnosis was reached when cultures were unrevealing, how a below-threshold sequencing result was weighed in the clinical context, and the host factors that may have predisposed to this infection.

## 2 Case presentation

An 83-year-old man underwent coronary stent implantation for angina at an outside hospital on 5 December 2024. Echocardiography on the day of PCI showed septal thickening, aortic valve degeneration, grade I diastolic dysfunction, normal left ventricular (LV) systolic function, and a small pericardial effusion, without pericardial adhesion or constrictive features. He recovered well and was maintained on aspirin, clopidogrel, rabeprazole, rebamipide, trimetazidine, and atorvastatin. He had prior stenting in 2017 and a CKD of 8 years’ duration (serum creatinine 140–160 μmol/L over the preceding 6 months), no hypertension or diabetes (HbA1c 6.3% on this admission), and no history of tuberculosis. He had a 15-year smoking history (quit >30 years ago) and denied alcohol use. He was independent in daily activities.

About 6 weeks after stenting (mid-January 2025), the patient developed cough and low-grade fever. Self-administered second-generation cephalosporins and moxifloxacin provided fluctuating relief. In late January, an outside hospital found leukocytosis and elevated C-reactive protein (CRP), and levofloxacin for presumed urinary infection did not resolve the fever. He was hospitalized on 2 February and received ticarcillin-clavulanate and then cefoperazone-sulbactam, but he continued to have nocturnal fevers (peak >38.5°C). A single draw (one side, two bottles) on 5 February grew coagulase-negative staphylococcus. The number of positive bottles was not available to us, and no further cultures were obtained there. He was transferred to our department on 8 February for a fever of unknown origin.

**Figure 1:**
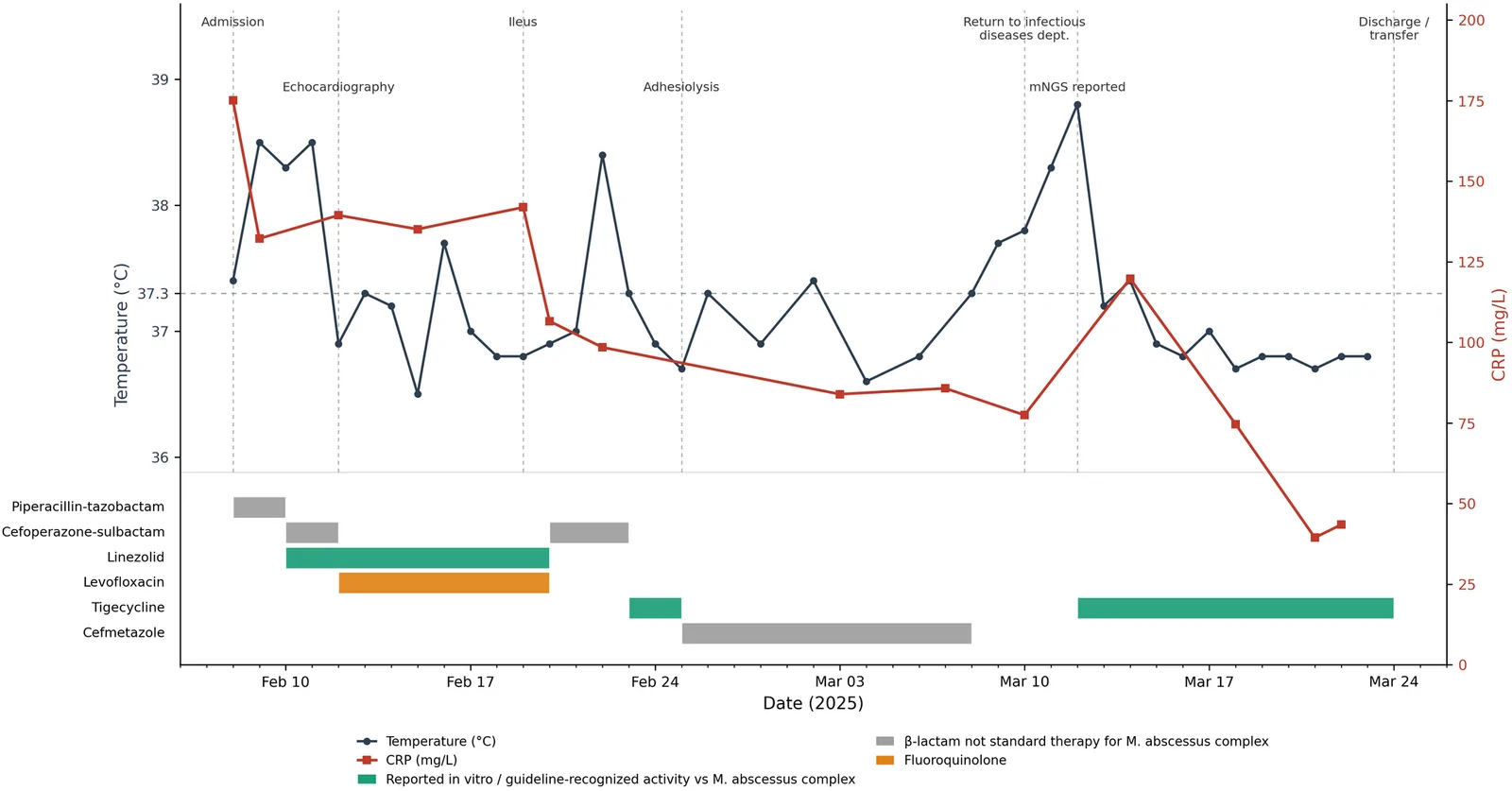
Daily maximum body temperature and C-reactive protein (CRP) during hospitalization, shown with antimicrobial therapy and key clinical events. Temperature and CRP values are from the clinical record; during the postoperative surgical period, temperature is shown at representative time points. The colored bars indicate the duration of each antimicrobial agent: green, agents with reported *in vitro* or guideline-recognized activity against the *Mycobacterium abscessus* complex (linezolid and tigecycline); gray, β-lactams that are not standard therapy for this organism (note that β-lactam activity varies by compound, and cefoxitin and imipenem, which were not used here, do have recognized activity); orange, fluoroquinolone. Dates are in 2025.

**Figure 2:**
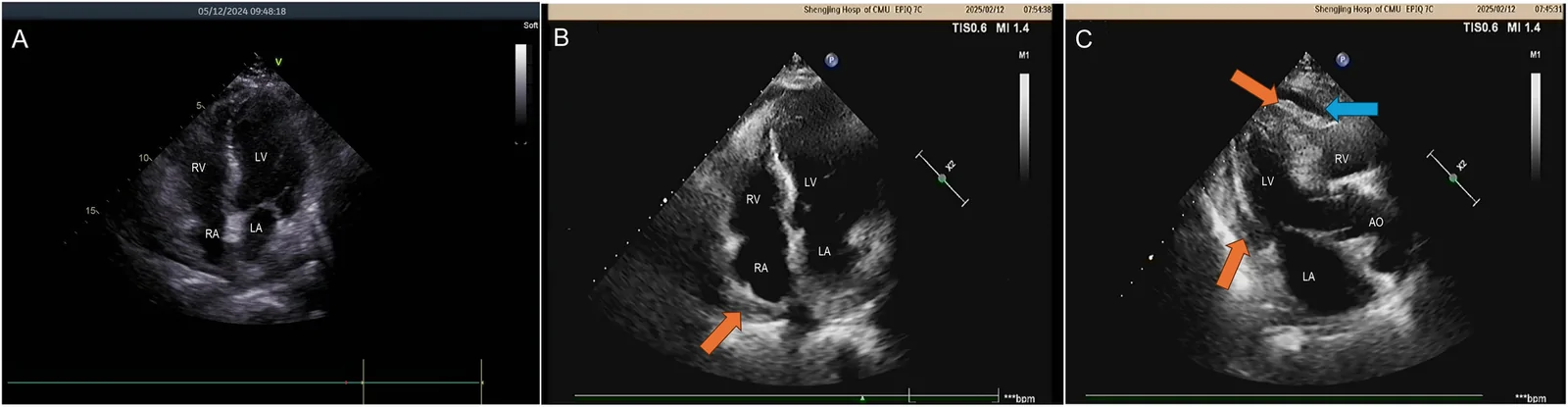
Transthoracic echocardiography before and after a percutaneous coronary intervention (PCI). **(A)** Before the PCI (5 December 2024): the pericardium is not thickened and shows no adhesion. **(B,C)** Current admission (12 February 2025): thickening of the visceral and parietal pericardium over the mid-to-lower left ventricle and the roof of the right atrium, with a hypoechoic layer within the pericardial space and focal visceral–parietal pericardial adhesion; a small pericardial effusion is seen elsewhere. The orange arrows indicate pericardial thickening; the blue arrows indicate the newly detected effusion. LV, left ventricle; RV, right ventricle; LA, left atrium; RA, right atrium; AO, aorta.

**Figure 3:**
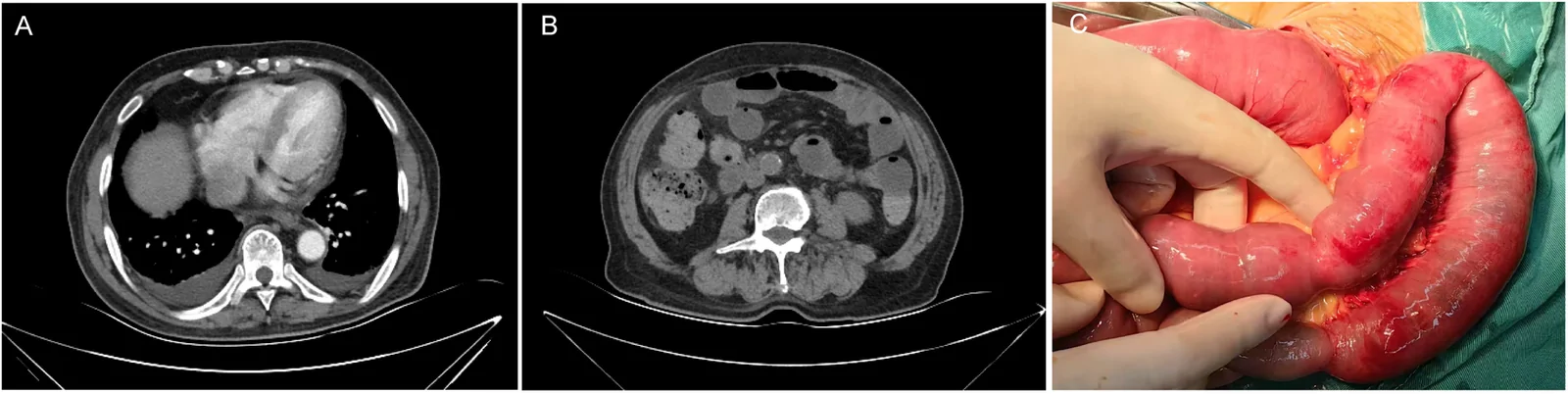
Imaging and intraoperative findings. **(A)** A chest CT on admission (9 February 2025): bilateral pleural effusions. **(B)** A whole-abdomen CT (18 February 2025): dilated small bowel with air–fluid levels, indicating mechanical small-bowel obstruction. **(C)** An intraoperative view obtained after adhesiolysis (25 February 2025), showing the previously herniated small-bowel segment with punctate hemorrhage, dusky discoloration, and mesenteric edema and congestion. No photograph was taken before adhesiolysis; panel C therefore documents bowel compromise and does not itself depict the omental adhesions or provide evidence of mycobacterial serosal fibrosis.

On admission, the patient was alert and hemodynamically stable, with coarse breath sounds, a regular heart rhythm, a soft non-tender abdomen, and bilateral lower-limb edema. An examination of the skin, including the previous arterial access site, showed no breakdown, abscess, draining sinus, or pseudoaneurysm, although the site was not reimaged. Temperature and CRP trends, with antimicrobial therapy and key events, are shown in Figure 1, and laboratory trends are provided in Table 1. Blood cultures were repeated twice after admission, each time from both arms (two bottles per draw), and were negative. A bone-marrow culture was also negative. Temperature fell and normalized after linezolid (0.6 g q12 h) with levofloxacin (0.25 g qd, 0.5 g loading dose), albumin, and supportive care. Echocardiography on 12 February showed focal visceral–parietal pericardial adhesion at the LV apical third with regional wall-motion restriction, a thickened echogenic pericardium over the right atrium, a diffuse small pericardial effusion (3–5 mm), and restricted biventricular diastolic filling, with normal LV systolic function. These features suggested early constrictive physiology, although strict echocardiographic criteria for constrictive pericarditis were not fulfilled. The pericardial adhesion was new compared with the preprocedure study (Figure 2). A chest computed tomography (CT) showed bilateral pleural effusions (Figure 3A), with inconspicuous parenchymal inflammation. T-SPOT.TB and repeated sputum acid-fast smears and mycobacterial cultures were negative. Thoracentesis failed because the effusion was too scant.

**Table 1:** Trends of major laboratory parameters during hospitalization (2025).

Parameter	8 February	12 February	15 February	19 February	22 February	3 March	10 March	14 March	18 March	20 March	22 March	Normal Range
WBC (×10⁹/L)	15.7	11	9.9	8.6	8.4	13.1	10.8	9.6	9.1	8.8	—	3.5–9.5
LY (×10⁹/L)	0.8	1.4	1.5	1.0	1.4	1.8	1.3	1.2	1.7	2.2	—	1.1–3.2
LY (%)	5.2	12.3	15.3	12.0	16.8	14.0	11.9	12.2	18.6	25.3	—	20–50
NE (%)	88.1	73.4	71.7	83.6	72.9	72.6	76	74.4	67.9	63.6	—	40–75
HGB (g/L)	99	90	89	88	85	64	69	80	88	100	—	130–175
PLT (×10⁹/L)	270	234	162	118	72	222	347	419	533	596	—	125–350
CRP (mg/L)	175.04	139.42	135.1	141.9	98.46	83.91	77.42	119.79	74.62	—	43.43	0–6
PCT (ng/mL)	0.366	0.287	0.366	0.309	1.01	1.03	0.405	0.357	0.354	—	—	<0.05
ESR (mm/h)	93	—	—	65	46	—	77	—	88	—	—	0–15
ALB (g/L)	24.3	22.4	27.4	30.9	29.1	23.2	23.7	28.5	31	—	—	40–55
ALT (U/L)	39	20	13	13	12	61	38	35	43	—	—	9–50
AST (U/L)	52	28	25	19	22	109	43	30	51	—	—	15–40
Tbil (μmol/L)	7.9	13.4	14.7	13.3	10.7	6.2	5.9	8.9	11.7	—	—	0–26
Cr (μmol/L)	184	184	210	250	260	158	163	188	220	242	226	57–111
eGFR (mL/min/1.73m^2^)	31	31	26	21	20	37	36	30	25	22	24	≥90
BNP (pg/mL)	425.4	467.5	227.2	119.8	79.4	—	392.8	133.9	97.7	—	—	0–154.7
INR	1.2	1.2	1.2	1.2	1.1	—	1.3	—	1.2	1.2	—	0.8–1.2
D-dimer (μg/mL)	3.5	4.14	2.92	3.47	3.4	—	2.35	—	1.62	1.29	—	0–0.5

WBC, white blood cell count; NE, neutrophils; HGB, hemoglobin; PLT, platelets; LY, lymphocytes; CRP, C-reactive protein; ESR, erythrocyte sedimentation rate; PCT, procalcitonin; ALB, albumin; ALT, alanine aminotransferase; AST, aspartate aminotransferase; Tbil, total bilirubin; Cr, creatinine; eGFR, estimated glomerular filtration rate (CKD-EPI 2021); BNP, B-type natriuretic peptide; INR, international normalized ratio. The ESR was measured on 9 and 19 February, 22 February, and 10 and 18 March; the value shown in the 8 February column was obtained on 9 February.

On 18 February, abdominal pain prompted a whole-abdomen CT, showing dilated, fluid-filled small-bowel loops with surrounding exudation and increased peritoneal effusion (Figure 3B). On 19 February, the patient developed vomiting with cessation of flatus and stool, and mechanical small-bowel obstruction was diagnosed. Linezolid and levofloxacin were stopped and cefoperazone-sulbactam (1.5 g q12 h) started. Fever recurred on 22 February, and tigecycline (50 mg q12 h) with intravenous immunoglobulin (5 g qd, 23–25 February) was given, but for only 2 days, as conservative management failed and laparotomy was performed on 25 February. Operative findings revealed approximately 500 mL of bloody peritoneal fluid and a dilated, fluid-filled small bowel. Adhesive bands between the greater omentum and the abdominal wall, with approximately 40–50 cm of small bowel herniated through and the distal bowel empty, identified the obstruction site. The bands were divided and ligated and the entrapped bowel released, restoring patency. The herniated segment showed punctate hemorrhage, a dusky color, and mesenteric edema and congestion but recovered peristalsis on stimulation, and therefore, no resection was performed (Figure 3C). A re-exploration found no perforation or tumor, with a smooth liver surface. Peritoneal fluid and omental tissue were not submitted for a microbiological or histological examination. Cefmetazole (2.0 g bid, 25 February–8 March) was given postoperatively, with persistent intermittent fever.

The patient’s fever remained uncontrolled, and further evaluation was undertaken on 10 March. pANCA and antimyeloperoxidase (MPO) antibody (>739.8 CU, negative < 20 CU) were strongly positive with ANA 1:160, whereas cANCA, PR3, rheumatoid factor, and antistreptolysin O were all negative. A urinalysis showed persistent hematuria (occult blood 3+; RBC 15-31/HPF) and proteinuria (1-2+; 24-h urinary protein 1.1 g). Creatinine levels rose from a baseline of 160–184 to a peak of 260 μmol/L, with the estimated glomerular filtration rate falling from approximately 31 to 20 mL/min/1.73 m^2^ (CKD stage G4) before making partial recovery. The erythrocyte sedimentation rate was persistently elevated (46–93 mm/h; reference 0–15). IgA was elevated and IgM low, with normal IgG (reference: IgA 0.7–4.0, IgG 7.0–16.0, and IgM 0.4–2.6 g/L). Lymphocyte subsets during acute illness showed marginally low CD3+ (648/μL; reference 690–2,540) and CD4+ (401/μL; reference 410–1,590) counts, with intermittently low absolute lymphocytes (0.7–2.2 × 10⁹/L; reference 1.1–3.2). Immunofixation, free light-chain *κ*/*λ* ratio (1,3)-β-D-glucan, galactomannan, and HIV antibody were all negative; serum IgG4 was not measured. Whole-body 18F-FDG PET/CT (13 February) showed no malignancy, with splenic and axial marrow uptake and a small renal-hilar node interpreted as reactive; bilateral renal cortical scarring was consistent with CKD. Bone marrow aspiration excluded myeloma.

Blood DNA and RNA mNGS detected *herpes simplex virus type 1* (HSV-1; four reads; “virus positive”) and the *Mycobacterium chelonae complex* at complex level (four reads), of which three were assigned at species level to *M. abscessus* (relative abundance 0.0059%), below the platform threshold (≥8 reads) and reported as “suspected bacterium.” Subspecies identification was not possible. Assay details, quality-control and negative-control results, thresholds, and read and coverage data are given in Supplementary Table S1. Given recurrent fever after stenting, pericardial and intra-abdominal adhesions, improvement during the administration of linezolid and tigecycline and relapse during the administration of β-lactams inactive against this organism, a clinically suspected diagnosis of *M. abscessus* infection supported by mNGS was made after consultation with a tuberculosis specialty hospital. Tigecycline was restarted on 12 March (100 mg loading dose, 50 mg q12 h). HSV-1 DNAemia was not confirmed by quantitative PCR, unavailable at our institution, and there were no orolabial, mucocutaneous, or neurological manifestations. Empirical ganciclovir (250 mg q12 h, 12–18 March) was given for possible reactivation, acyclovir not being available in our formulary, with immunoglobulin (5 g qd, 14–20 March) for support. HSV-1 was not considered the cause of the serosal findings. Temperature normalized and the patient’s condition improved. The consultants recommended a four-drug regimen (tigecycline or omadacycline, clarithromycin, linezolid, and clofazimine, renally adjusted) and accepted transfer. The patient was discharged and transferred on 24 March. However, he was subsequently lost to follow-up. Therefore, further treatment, the final outcome, and the patient's perspective could not be obtained. Written informed consent for publication had been obtained from the patient during hospitalization.

## 3 Discussion

This patient developed pericardial adhesion, bilateral pleural effusions, and dense omental adhesions within a short period, with no history of abdominal surgery. Such synchronous involvement of more than one serosal compartment pointed to a systemic process, and, taken together with the febrile course and the response to antimicrobial therapy, we judged infection to be the most likely explanation and treated the patient accordingly. It should be acknowledged that this attribution rests on clinical inference: neither pericardial fluid nor omental tissue was sampled, and thoracentesis was unsuccessful because the effusion was too scant, and therefore, no histological or microbiological corroboration was available. CKD, hypoalbuminemia (22–24 g/L), edema, and an elevated B-type natriuretic peptide may also have contributed to serosal fluid. A small pericardial effusion predated the PCI, and the bloody peritoneal fluid may, in part, reflect mechanical obstruction itself. *M. abscessus* infection after coronary stenting has been reported previously, but predominantly as endocarditis: a review summarized 25 cases after cardiac catheterization or intervention, all with valvular vegetations (13); disseminated infection after peripheral vascular stenting has also been described (14, 15). In contrast, our patient had no vegetations, and the abnormalities were confined to the serosal surfaces. A PubMed search for case reports of *M. abscessus* infection, last updated 31 May 2025, identified no comparable polyserositis presentation after coronary stenting.

The pericardial timeline was informative: the preprocedure study showed only a small effusion without adhesion, whereas the study 2 months later showed adhesion with changes suggesting early constrictive physiology. Because *Mycobacterium tuberculosis* is the classic cause of constrictive pericarditis (16), the negative T-SPOT.TB and sputum studies made tuberculous involvement less likely. However, these target *M. tuberculosis*, have limited sensitivity for extrapulmonary disease, and are insensitive to NTM, and hence, tuberculous pericarditis cannot be excluded. The new adhesion and diastolic restriction were consistent with evolving constrictive physiology, although the inferior vena cava showed normal respiratory variation, and multiparametric assessment, cardiac CT or MRI, and invasive hemodynamics were not performed, and thus, established constrictive pericarditis could not be diagnosed. NTM pericarditis is rarely reported. Figueiredo et al. (17) recently described NTM pericarditis with polyserositis that somewhat resembles our case.

Abdominal involvement is also noteworthy: dense omental–parietal adhesions and bloody peritoneal fluid were found, although the patient had no prior abdominal surgery or malignancy. Rouhani and Adunuri (18) reported *M. abscessus* peritonitis complicated by encapsulating peritoneal sclerosis with small-bowel obstruction, resembling our case, although that patient was on peritoneal dialysis. Both suggest that *M. abscessus* can cause chronic serosal inflammation with fibrosis and adhesion, consistent with mycobacterial granulomatous inflammation (19, 20). The operation was undertaken urgently for mechanical small-bowel obstruction, and peritoneal fluid and omental tissue were not submitted for a microbiological or histological examination. No material remained for retrospective testing. The association between the adhesions and *M. abscessus* therefore remains an inference. In retrospect, intraoperative sampling would have been informative, and we would now advocate that serosal fluid and tissue be sent for mycobacterial culture and histology whenever a patient with unexplained fever undergoes abdominal surgery.

mNGS was pivotal: with cultures repeatedly unrevealing, it was the only investigation that pointed to a specific pathogen, and it illustrates both the value and the limits of the technique. At the referring hospital, a single blood-culture draw grew coagulase-negative *Staphylococcus*. The number of positive bottles, species identification, and time to positivity were unavailable to us, and no repeat cultures were taken there. Given negative repeat blood and bone-marrow cultures after admission, no vegetations on echocardiography, and a polyserositis picture unexplained by staphylococcal bacteremia, contamination was considered most likely, although endovascular infection was not formally evaluated by transesophageal echocardiography. The three species-level *M. abscessus* reads fell below the threshold (≥8), whereas HSV-1 with four reads met the viral threshold (≥3), reflecting intrinsic differences in detection efficiency. In this patient, a low read count was not unexpected: the mycolic-acid-rich cell wall impedes nucleic-acid release, and prolonged antibiotic exposure had suppressed bacterial load (19). Read together with the clinical course and the treatment response, we considered the finding informative. A low-read signal may equally arise from contamination, taxonomic misclassification within the *M. chelonae–abscessus* group, or clinically irrelevant detection; species assignment rested on database alignment, and three reads with negligible genome coverage cannot guarantee reliable discrimination from closely related members. Repeat mNGS was not undertaken, given the cost of the assay and the clinical course. We therefore did not treat the read count itself as diagnostic but used it as a pointer to a likely etiology, with the working diagnosis resting on the overall clinical assessment rather than on sequencing alone (9, 10, 21).

The treatment response was the second supportive evidence of the working diagnosis. Temperature did not improve during the administration of the initial β-lactams (ticarcillin-clavulanate and cefoperazone-sulbactam at the referring hospital, and piperacillin-tazobactam after admission). It normalized within a week of adding linezolid, recurred when linezolid was stopped for ileus and cefoperazone-sulbactam was resumed, and normalized again after tigecycline was restarted. This pattern of relapse on withdrawal corresponds to the intrinsic resistance profile of *M. abscessus* and was one of the main grounds for our working diagnosis. It is not, however, specific evidence: both agents are active against many other organisms, and surgery, supportive care, and spontaneous fluctuation of fever may all have contributed, and therefore, the pattern alone cannot establish the etiology. β-lactam activity also varies by compound: cefoxitin and imipenem have recognized activity against the complex, whereas the agents used here do not represent standard therapy (2, 22). For the regimen proposed at transfer, no isolate was available, and therefore, subspecies determination, susceptibility testing, *erm*(41) characterization, and *rrl* testing could not be performed, and clarithromycin was included empirically. Amikacin was avoided because of stage G4 CKD and the risks of nephrotoxicity and ototoxicity at age 83. Imipenem and cefoxitin were not proposed, although guideline regimens often incorporate them (23). Because treatment after transfer was never documented, we cannot state whether it was implemented, tolerated, or effective. Transaminases peaked on 3 March [alanine aminotransferase (ALT) 61, aspartate aminotransferase (AST) 109 U/L] during the administration of cefmetazole rather than tigecycline or linezolid.

Several factors may have contributed to age- and CKD-associated immune dysfunction. Immunosenescence at 83 reduces T-cell diversity and macrophage phagocytosis (6, 24), and CKD impairs neutrophil chemotaxis and T-cell function (7). The abnormalities here were nevertheless marginal: CD3+ and CD4+ counts fell only slightly below the local lower limits and were obtained during acute illness, when transient lymphopenia is common. These values, with a low IgM, indicate immune fragility rather than a defined immunocompromised state, and low-abundance HSV-1 detection without clinical disease does not itself establish meaningful impairment of cellular immunity. NTM infection has been associated with cardiac surgery and intervention, including a postoperative *M. abscessus* cluster, device infection, bioprosthesis-associated *M. chelonae* endocarditis, and extrapulmonary disease in heart-transplant recipients (25–28).

Strongly positive MPO-ANCA (>739.8 CU; reference, negative <20 CU) with pANCA and ANA raised ANCA-associated vasculitis as the first differential consideration. The patient, however, had none of the extrarenal features of systemic vasculitis (no ocular inflammation, oral ulceration, rash, purpura, arthritis, mononeuritis multiplex, or pulmonary hemorrhage). On rheumatology consultation, the advice was to treat the presumed infection first and not to attribute the findings to vasculitis before infection and malignancy had been excluded, with high-dose corticosteroids deferred in view of his age. Mycobacterial infection can itself induce MPO-ANCA (11, 12), and microscopic polyangiitis after pulmonary NTM disease has been reported (29). On this basis, we regarded the autoantibodies as an infection-associated, non-specific phenomenon and did not treat the patient for primary vasculitis. PET/CT showed no malignancy, and nephrology attributed the acute rise in creatinine levels to postoperative and drug-related acute kidney injury superimposed on 8 years of CKD; a plasma-cell disorder was excluded. A renal biopsy was not pursued given his age and clinical instability, and loss to follow-up precluded serial ANCA measurement, and therefore, this interpretation rests on the clinical picture rather than on histology or antibody trajectory.

Other diagnoses were considered but were not supported. PET/CT showed no malignancy, the marrow was unremarkable, and no tumor was detected upon laparotomy, arguing against peritoneal carcinomatosis or lymphoma. Negative (1,3)-β-D-glucan and galactomannan made invasive fungal infection unlikely. Endovascular bacterial infection was improbable, with repeatedly negative blood and bone-marrow cultures and no vegetations on echocardiography. Drug-induced fever was unlikely, as fever preceded hospital treatment and defervescence occurred during, and not after, antimicrobial therapy. Postoperative or ischemic adhesions cannot account for the pericardial changes, which preceded surgery, and nothing in the clinical picture suggested IgG4-related disease, although serum IgG4 was not measured. Two alternatives could not be formally excluded: tuberculous serositis, because T-SPOT.TB and sputum testing target *M. tuberculosis* and are insensitive to extrapulmonary disease, and misclassification within the *M. chelonae-abscessus* group. Against tuberculosis, however, the patient had no exposure history and did not defervesce on regimens containing agents with antituberculous activity, whereas he responded to linezolid and tigecycline.

The main shortcoming of this report is the lack of orthogonal microbiological confirmation. Dedicated mycobacterial blood culture is not available at our institution, and conventional blood culture systems are not designed to recover mycobacteria. At the time of surgery, mycobacterial infection had not yet been suspected, and therefore, peritoneal fluid and tissue were not retained, and no material was subsequently available for targeted PCR, acid-fast staining, or mycobacterial culture. For the pericardial findings, cardiac CT or MRI and invasive hemodynamic assessment were not undertaken in view of the patient's age and clinical condition, and therefore, the echocardiographic changes could only be described as suggestive of early constrictive physiology. Thoracentesis failed because the pleural effusion was scant, and transesophageal echocardiography and renal biopsy were likewise not performed. No environmental investigation was carried out at the referring facility, and thus, a procedure-related route of acquisition can be neither supported nor excluded. Finally, the patient was lost to follow-up after discharge, and hence, subsequent treatment, the ANCA trajectory, and follow-up imaging are unavailable.

## 4 Conclusion

We reported a rare case of a patient who presented with suspected *M. abscessus*-associated polyserositis following coronary stenting, with synchronous pericardial, pleural, and peritoneal abnormalities. With cultures repeatedly negative, the working diagnosis was reached by integrating a below-threshold mNGS result with the clinical course and the treatment response, and it guided therapy that was followed by defervescence and clinical improvement, although microbiological confirmation was not obtained. NTM infection should be considered in older patients with unexplained fever after cardiovascular intervention, particularly with age- and CKD-associated immune dysfunction. Low-abundance mNGS results deserve careful interpretation rather than automatic rejection, weighed against contamination and misclassification. NTM can induce non-specific MPO-ANCA positivity that must be distinguished from primary vasculitis.

## Data Availability

The original contributions presented in the study are included in the article/Supplementary Material, further inquiries can be directed to the corresponding author/s.
