# Supplementary material for: Case Report: Suspected Mycobacterium abscessus–associated polyserositis following coronary stenting, diagnosed with the aid of metagenomic next-generation sequencing
Source: Front Med (Lausanne). 2026 Sep 9;13:1910932. doi: 10.3389/fmed.2026.1910932 (PMC13597330; doi:10.3389/fmed.2026.1910932)
Supplement: Supplementary file 1 [file Table1.docx]

**Supplementary Table S1.** Details and quality metrics of the metagenomic next-generation sequencing (mNGS) assay.

| **Item** | **Detail** |
| --- | --- |
| Assay | PIseq DNA + RNA co-detection metagenomic next-generation sequencing |
| Laboratory | WillingMed Technology (Beijing) Co., Ltd. |
| Sample type | Peripheral blood (EDTA), 5 mL; collected 10 March 2025; sample quality unremarkable |
| Host depletion | pathoXtract host nucleic-acid removal |
| Quality control | Positive control, negative control, and internal control all passed; GC content 42%; Q30 93.4% |
| Negative (no-template) control | Passed, with no evidence of background contamination |
| Positivity thresholds (laboratory-defined) | Bacteria ≥ 8 species-level reads; viruses ≥ 3 species-level reads |
| *Mycobacteroides abscessus* | Genus *Mycobacterium chelonae* complex, 4 reads; species *M. abscessus*, 3 reads; aligned length 196 bp; relative abundance 0.0059%; below the bacterial threshold; qualitative call "suspected" |
| Herpes simplex virus type 1 | 4 reads; aligned length 245 bp; at/above the viral threshold; qualitative call "positive" |
| Antimicrobial-resistance and virulence genes | None detected |
| RNA viruses / fungi / parasites | None detected |

**Notes on interpretation and limitations.**

The assay is a shotgun metagenomic method. Quality-control metrics (positive, negative, and internal controls all passed; Q30 93.4%) indicate acceptable sequencing quality, and the negative control showed no evidence of reagent or workflow contamination. However, this does not exclude contamination introduced at sampling or low-level environmental background, which remains a consideration for any low-read result. The assay is a laboratory-developed test performed by the vendor's laboratory, which is not formally accredited (e.g., ISO 15189). Results are intended for research and clinical reference.

Several limitations should be acknowledged. First, *M. abscessus* was detected at very low abundance (3 species-level reads; 196 bp of aligned sequence; genome coverage 0.00%), below the laboratory's positivity threshold, and was reported only as a "suspected" organism within the assay's technical grey zone. Second, species-level assignment within the *Mycobacterium chelonae-abscessus* complex was based on the laboratory's reference-database alignment; given the low read count and negligible genomic coverage, reliable discrimination of *M. abscessus* from closely related complex members cannot be guaranteed from these data alone. Third, we cannot confirm from the report whether the three species-level reads mapped uniquely to *M. abscessus*, and repeat mNGS was not undertaken, given the cost of the assay and the patient's clinical course. Fourth, no orthogonal confirmation was available: dedicated mycobacterial blood culture is not performed at our institution, conventional blood cultures (not designed to recover mycobacteria) were negative, sputum testing directed at *M. tuberculosis* was negative and is insensitive to nontuberculous mycobacteria, and no archived material was available for retrospective testing.

Accordingly, the mNGS result is interpreted as a below-threshold signal that pointed to a specific pathogen when conventional cultures were unrevealing, rather than as microbiological proof. *M. abscessus* infection is reported as a suspected (working) diagnosis, reached by integrating the sequencing result with the clinical course, treatment response, and specialist consultation.
